# Lévy foraging patterns of rural humans

**DOI:** 10.1371/journal.pone.0199099

**Published:** 2018-06-18

**Authors:** Andy Reynolds, Eliane Ceccon, Cristina Baldauf, Tassia Karina Medeiros, Octavio Miramontes

**Affiliations:** 1 Rothamsted Research, Harpenden, United Kingdom; 2 Centro Regional de Investigaciones Multidisciplinarias, UNAM, Cuernavaca, Mexico; 3 Biological and Health Sciences Centre, Federal Rural University of Semiarid Region (UFERSA), Mossoró, Brazil; 4 Instituto de Fisica & C3, UNAM, Mexico City, Mexico; 5 Applied Mathematics and Statistics, EIAE, Universidad Politécnica de Madrid, Madrid, Spain; University of Plymouth, UNITED KINGDOM

## Abstract

Movement patterns resembling Lévy walks, often attributed to the execution of an advantageous probabilistic searching strategy, are found in a wide variety of organisms, from cells to human hunter-gatherers. It has been suggested that such movement patterns may be fundamental to how humans interact and experience the world and that they may have arisen early in our genus with the evolution of a hunting and gathering lifestyle. Here we show that Lévy walks are evident in the Me’Phaa of Mexico, in Brazilian Cariri farmers and in Amazonian farmers when gathering firewood, wild fruit and nuts. Around 50% of the search patterns resemble Lévy walks and these are characterized by Lévy exponents close to 1.7. The other search patterns more closely resemble bi-phasic walks. We suggest potential generative mechanisms for the occurrence of these ubiquitous Lévy walks which can be used to guide future studies on human mobility. We show that frequent excursions and meanderings from pre-existing trails can account for our observations.

## Introduction

In recent years there has been an accumulation of evidence that a wide variety of organisms have movement patterns resembling Lévy walks (sometimes called Lévy flights in the biological and ecological literature) [1–15]. Lévy walks alternate clusters of many short steps with longer steps between them, creating fractal movement patterns that have no characteristic scale. The self-similar, fractal properties of Lévy walks can be advantageous when randomly searching, and as a result it has been hypothesised that there could be natural selection for Lévy walks [16,17]. Lévy walk movement patterns in human hunter-gatherers were first reported to exist in the Dobe Ju/’hoansi in the Kalahari Desert in Botswana and Namibia [18] and later, in a more detailed study, in the Hadza of northern Tanzania [9], prompting the suggestion that Lévy search patterns may be fundamental to how individuals experience and interact with the world across a wide range of ecological contexts [9]. Indeed, it is tempting to speculate that Lévy walking arose early in our genus with the advent of hunting and gathering, playing an important role in the evolution of human mobility [9].

In this study we determine how common Lévy walk movement patterns are among three rural populations and we propose hypotheses about processes that may explain such patterns. We examined individual movement patterns from three different and unrelated rural groups, among Me’Phaa farmers searching and collecting firewood in the forests of “La Montaña de Guerrero” in southern Mexico [19], among Brazilian savannah farmers searching for non-cultivated wild fruit in the Chapada do Araripe in Brazil, and among Brazilian Rondonia Amazonian farmers foraging for edible seeds in the rain forest. The people foraging in these areas knew where to find the best trees and knew the best way to reach to them along trails and physical features. These movements are therefore non-random.

We find that Lévy walks, of the kind seen in the foraging of the Hadza hunter-gatherers in Tanzania, are ubiquitous and characterise movements along pre-existing trails and topographical features which the farmers tend to follow.

We suggest interrelated, simple, generative mechanisms for the emergence of these Lévy walks along pre-existing trails and thereby advance mechanisms to potentially explain the origins of Lévy walk foraging patterns in rural humans.

## Methods

All participants in Mexico and Brazil volunteered freely and informed consent was obtained from all subjects. Observations performed did not involve retrieval of any human biological material such as tissue or fluids and no experimental procedure of a psychological nature was performed.

Rural volunteers in Mexico and Brazil participated in an unsupervised observation season designed to GPS record and track walking trajectories in the forest while performing foraging activities. In Mexico, 20 male participants came from the Me’Phaa ethnic group (which is regarded as a very low income group) in the state of Guerrero, Mexico [19] whose main economic activity is the low-scale cultivation of *Hibiscus sabdariffa* flower. The Me’Phaa collect fallen firewood as domestic energy supply. These gathering activities are not done on a daily basis but are unscheduled and done occasionally under demand and are performed individually. The Me’Phaa foraging trips were recorded while individuals searched for fuelwood scattered in the floor area of the surrounding forests. The observation season took place between April and December 2010. Due to the unsupervised nature of the study, some individuals used the loggers wrongly and registered all their movements during the day rather than just their foraging movements. 8 individuals returned good data.

Twenty male Brazilian volunteers came from farmers in the Cariri region of the state of Ceara in Brazil [20], a very low income region located in the northeast of this country. Cariri peasant walks were recorded while individuals searched and collected Pequi (*Caryocar coriaceum*) fruits in the trees of the Araripe National Forest (Chapada do Araripe). Pequi gathering goes typically from February until May each year when foragers perform almost daily foraging excursions into the savannah. All fruit collected is sold in local markets. In the other months of the years Cariri farmers engage in low-scale agriculture and livestock raising. The unsupervised observation season took place between January and March 2014. 6 individuals returned ‘good’ data corresponding to foraging excursions. The other individuals returned data corresponding to non-foraging activities such as trips to the cities and to walking inside their villages. These data were not used.

Eight individuals (7 males and 1 female) from the Amazonian state of Rondônia volunteered for this study. They are farmers whose families came from past rubber tappers (seringueiros) in the Amazon rainforest and they live in the neighbourhood of the Extractive Reserve (RESEX) Rio Ouro Preto. Today they are engaged in agriculture practices, mostly cultivating M*anihot esculenta* (cassava or mandioca). Since these are very low income people, they search for and gather fallen Brazilian nuts or Castanha do Pará (B*ertholletia excelsa*) seeds as a secondary economic activity. The nuts are then sold in Brazilian and Bolivian markets. The observation season took place between December 2016 and January 2017. 6 individuals returned good foraging data.

**Fig 1:**
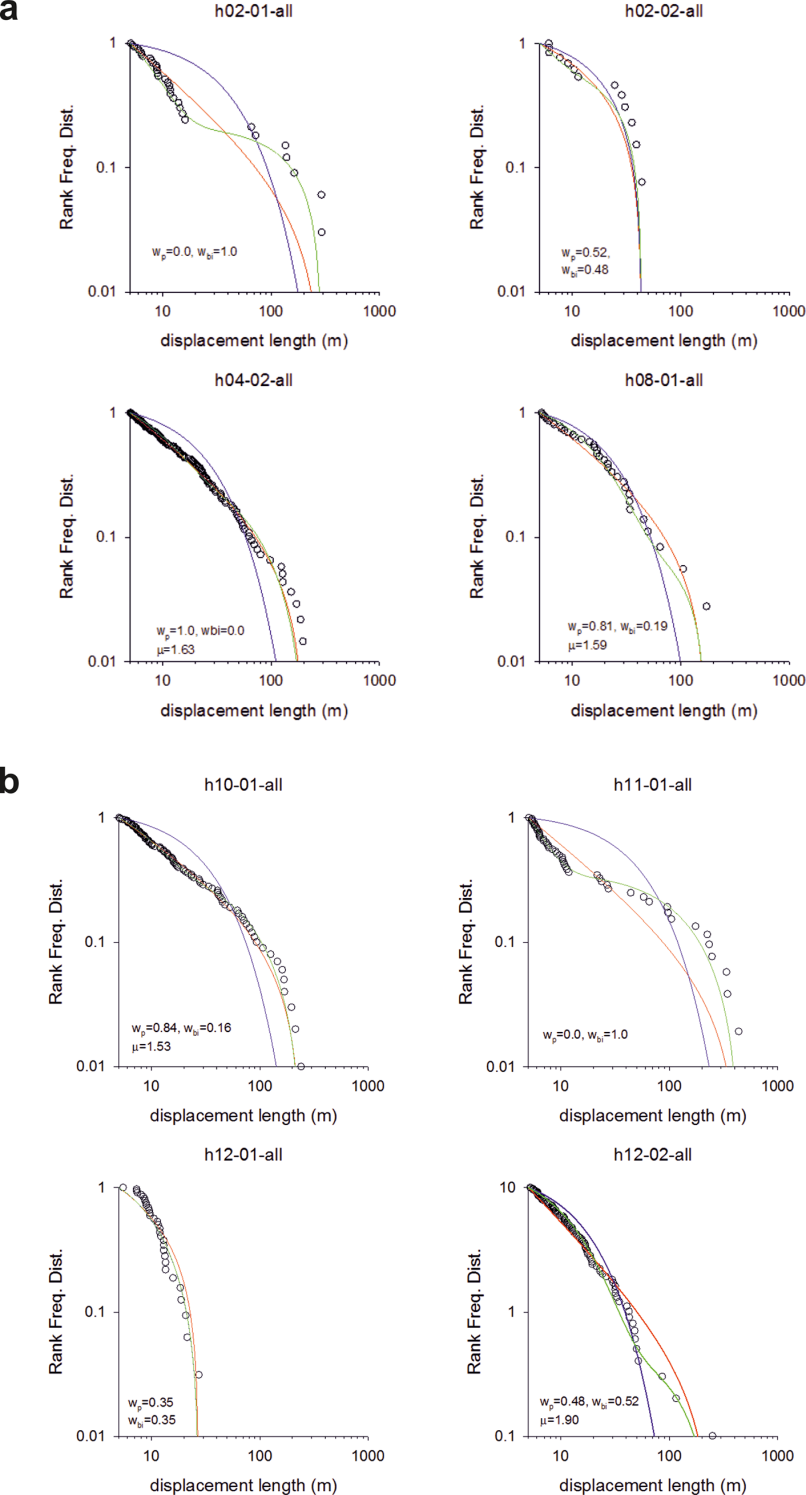
Fittings to the step length distribution for 8 individual Me’Phaa farmers searching for firewood. Rank frequency plots of the step lengths are shown (o) together with fits to power-law (red-lines), exponential (blue lines) and bi-exponentials (green lines). The Akaike weights, w_p_ and w_bi_, for the power-law and bi-exponential fits are shown together with the maximum likelihood estimates for the power-law (Lévy) exponents, μ. The numbers of steps before application of the method of Tromer et al. [21] are 247, 92, 455, 183, 258, 235, 77 and 239. After application of the method of Tromer et al. [21] which combines shorter steps into larger steps the numbers of steps are 33, 13, 138, 36, 100, 52, 32 and 99.

**Fig 2:**
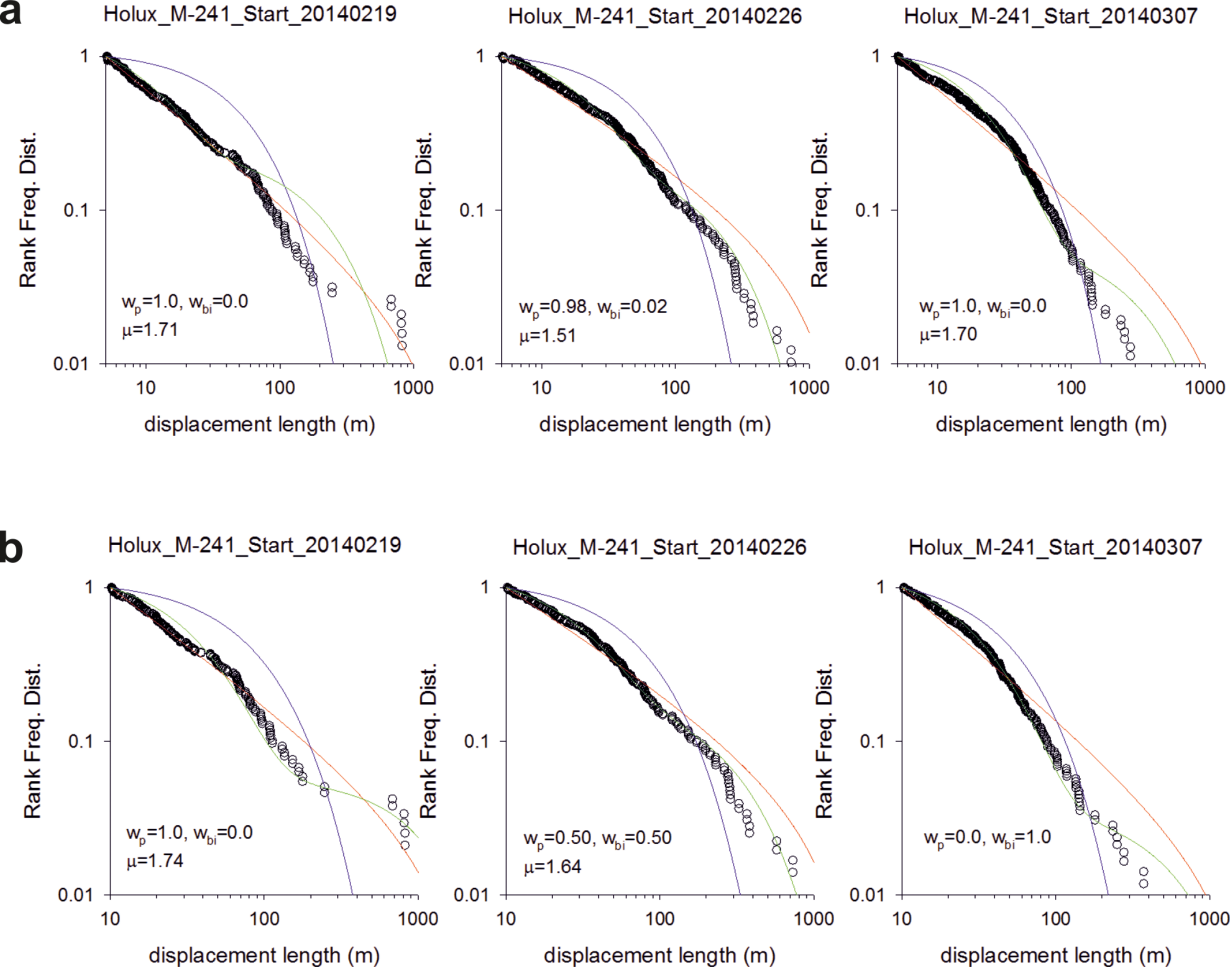
Fittings to the step length distribution for 6 Cariri Brazilian farmers searching for fruit. Rank frequency plots of the step lengths are shown (o) together with fits to power-law (red-lines), exponential (blue lines) and bi-exponentials (green lines). The Akaike weights, w_p_ and w_bi_, for the power-law and bi-exponential fits are shown together with the maximum likelihood estimates for the power-law (Lévy) exponents, μ. Outlying displacements due to initial bouts of cycle riding have not been removed prior to the analysis. The numbers of steps are 237, 357, 422, 380, 487 and 619.

**Fig 3:**
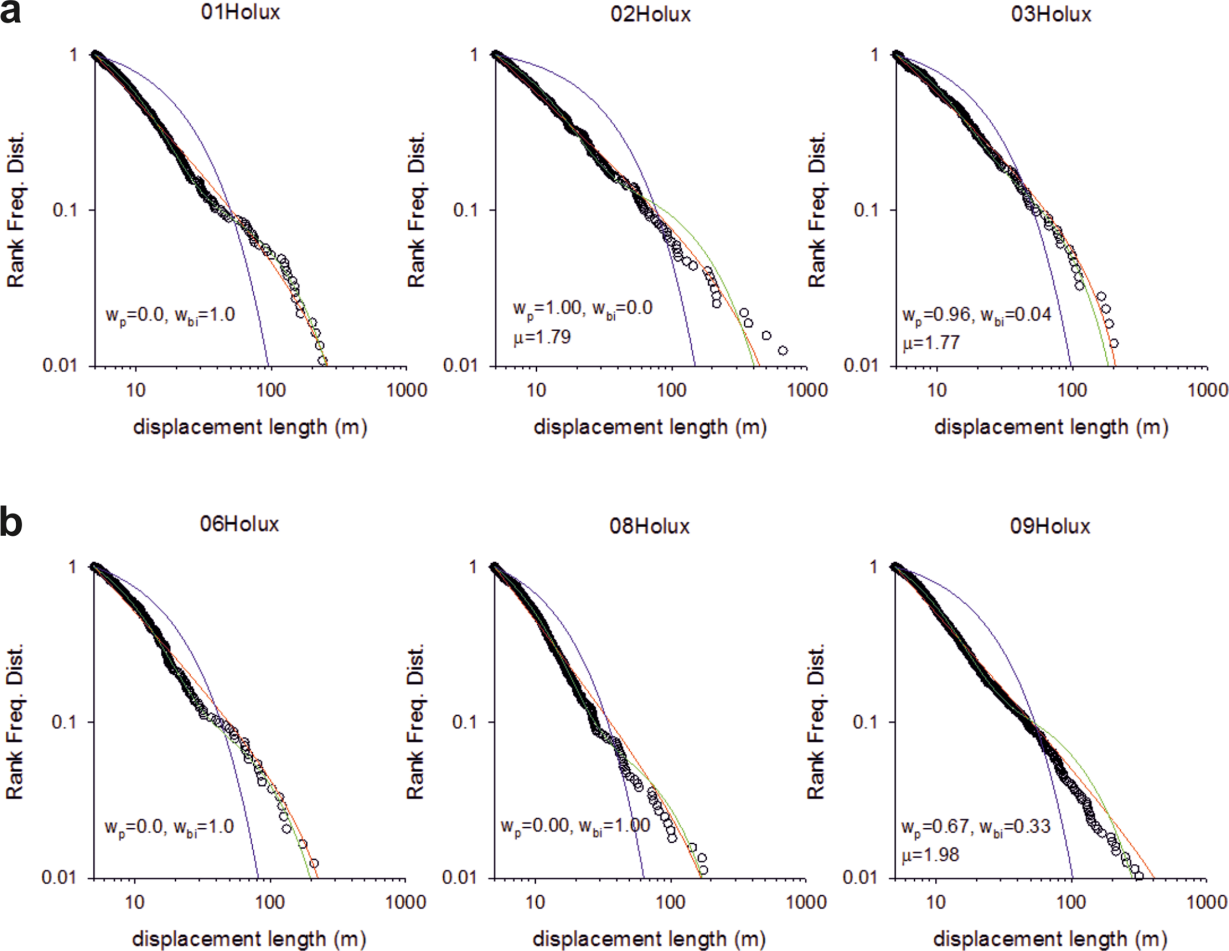
Fittings to the step length distribution for 6 Amazonian farmers searching for Brazilian nuts. Rank frequency plots of the step lengths are shown (o) together with fits to power-law (red-lines), exponential (blue lines) and bi-exponentials (green lines). The Akaike weights, w_p_ and w_b_i, for the power-law and bi-exponential fits are shown together with the maximum likelihood estimates for the power-law (Lévy) exponents, μ. The numbers of steps are 368, 317, 214, 241, 444 and 871.

In summary, we obtained 8 trajectories for the Me’Phaa, 6 for the Brazilian farmers and 6 for the Cariri. The statistics and model fits are shown in Figs 1–3.

All individuals in both Mexico and Brazil were trained to operate the GPS data loggers (Holux model M-241, by Holux Technology Inc. Hsinchu, Taiwan) in the field without supervision or accompaniment so that foraging activities were not externally influenced. The GPS datum was set to the standard WGS84. Devices were set to start recording after being switched on, and as soon as a valid fix was found. After the observation season, 35 foraging trajectories were recovered in Mexico, 29 in the Cariri savannah in Brazil and 23 in the Amazon forest in Brazil. Where several trajectories were recorded for an individual, only the first one was analysed to avoid any potential issues with duplication, i.e., with individuals repeating their movement patterns. Each walk was recorded with a 10 second time resolution and typically contained around 950 geoposition measurements each. No problems of satellite signal loss were identified.

To facilitate the identification of movement patterns resembling Lévy walks, we extracted sequences of ‘steps’ from each trajectory. This was done using the approach of Humphries et al. [6] in which the movement patterns are first projected onto the *x*- and *y*-axes to create two one-dimensional movement patterns for each individual. Humphries et al. [6] showed that the projection of a Lévy walk is itself a Lévy walk and that projection does not result in non-Lévy walks being misidentified as Lévy walks or vice versa. Turns in these projections can then be identified in an unambiguous way as occurring where the direction of travel changes. The projection method is now used widely because without projection turns can only be identified by making reference to arbitrarily defined critical-turning angles [6]. Analysis outcomes do not change significantly when, following Tromer et al. [21], the projection method is extended to account for noise in the data. Power-laws and competing, biologically-plausible models (exponential, bi-exponential, tri-exponential distributions) were fitted to the step-length distributions. Power-laws are indicative of true Lévy walks, exponentials are null models of the movement patterns indicative of random walking and multi-exponentials (mixtures of exponentials) are indicative of multiphasic searching. Fittings were performed by maximum likelihood methods [22] and the best model distribution was identified using the Akaike information criterion [23]. Distributions were truncated below 5 m (the length of the shortest resolvable step) and above the longest recorded displacement for each individual.

Our observations (step-length distributions) and theoretical expectations were compared with simulation data for the length of excursions from feature following. Excursions from direct movements (e.g., trail/feature following) were simulated using a simple discrete random walk model. At each time-step incremental displacements in the direction of the trail were drawn at random from an exponential distribution with mean 1 (arbitrary units a.u.). Incremental displacements in directions orthogonal to the trail were also exponentially distributed with mean 1 (a.u.). As a consequence simulated foragers tend to drift in the direction of the trail following artificial fluctuations (Figure A in S1 File). Simulation data was obtained for the net displacements made during off-trail excursions. Simulation data and empirical data were analysed in the same way. These predictions hold true generally because the decomposition into parallel and orthogonal movements is independent of the trail shape and because model predictions are constrained by the Sparre Andersen theorem [13,14]. This theorem dictates that the distribution of the durations of the longest excursions will have a 3/2 power-law tail.

## Results

**Fig 4:**
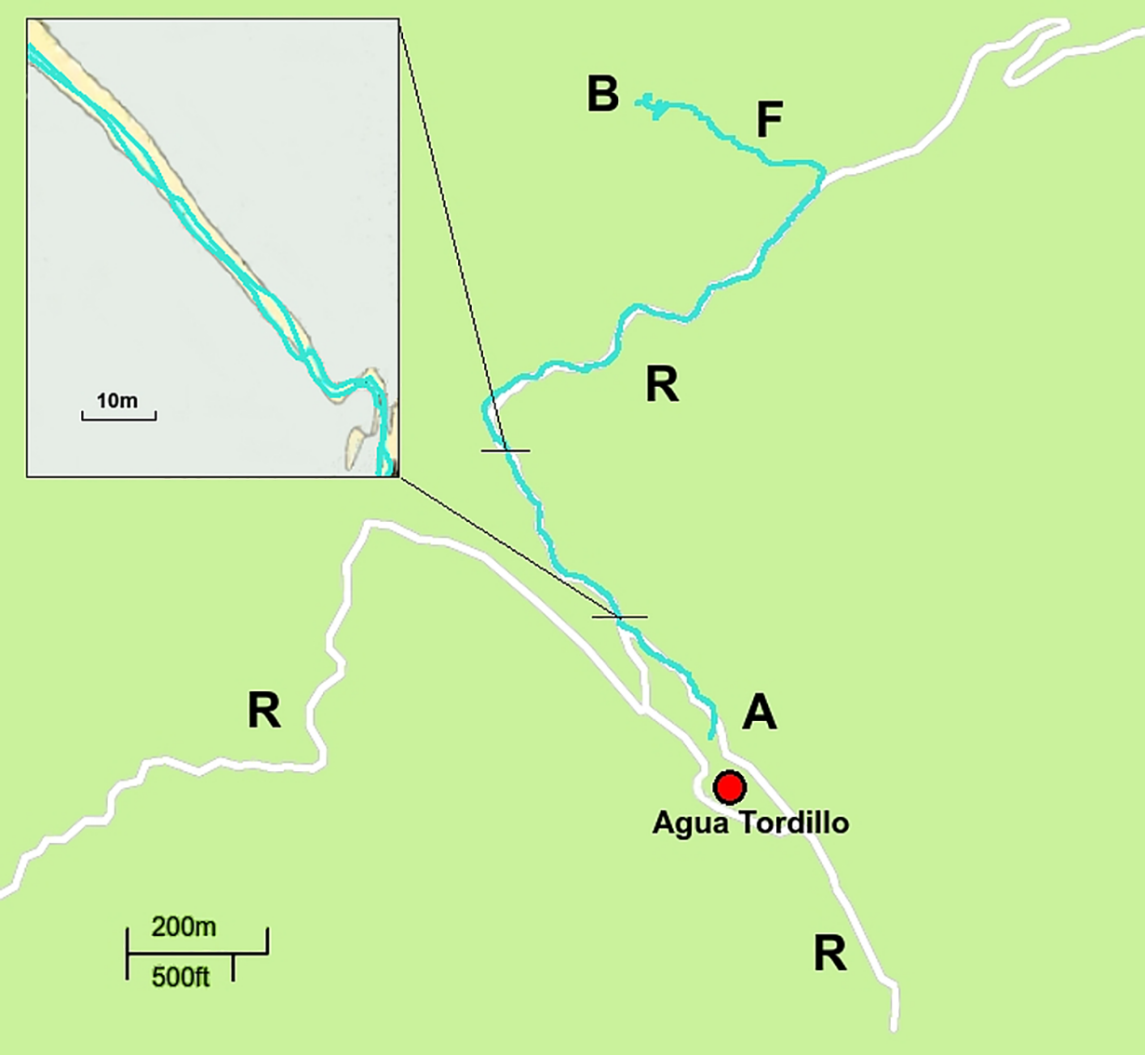
Trail following by the Me’Phaa out of Agua Tordillo small village. This is a typical example of a GPS recorded trajectory followed by an individual while executing a firewood searching exploration in the Guerrero Mountains in Mexico. The full trajectory is depicted overlaid in the map showing the main dirt roads used for walking. A is the start of the trajectory while B marks its end and the site where most of the wood search and collection has taken place. Once the individual goes off the dirty road (R) near the end of the trajectory, he goes into the woods following a network of footpaths (F). In the inset, a section of the outward and inward walking trajectory, as registered by the GPS, is shown overlaid over the almost straight line geometry of the dirt road segment. Notice the walker meandering across this bounded terrain feature. Map elaborated from our own GPS records. See S1 File for more details about this figure.

**Fig 5:**
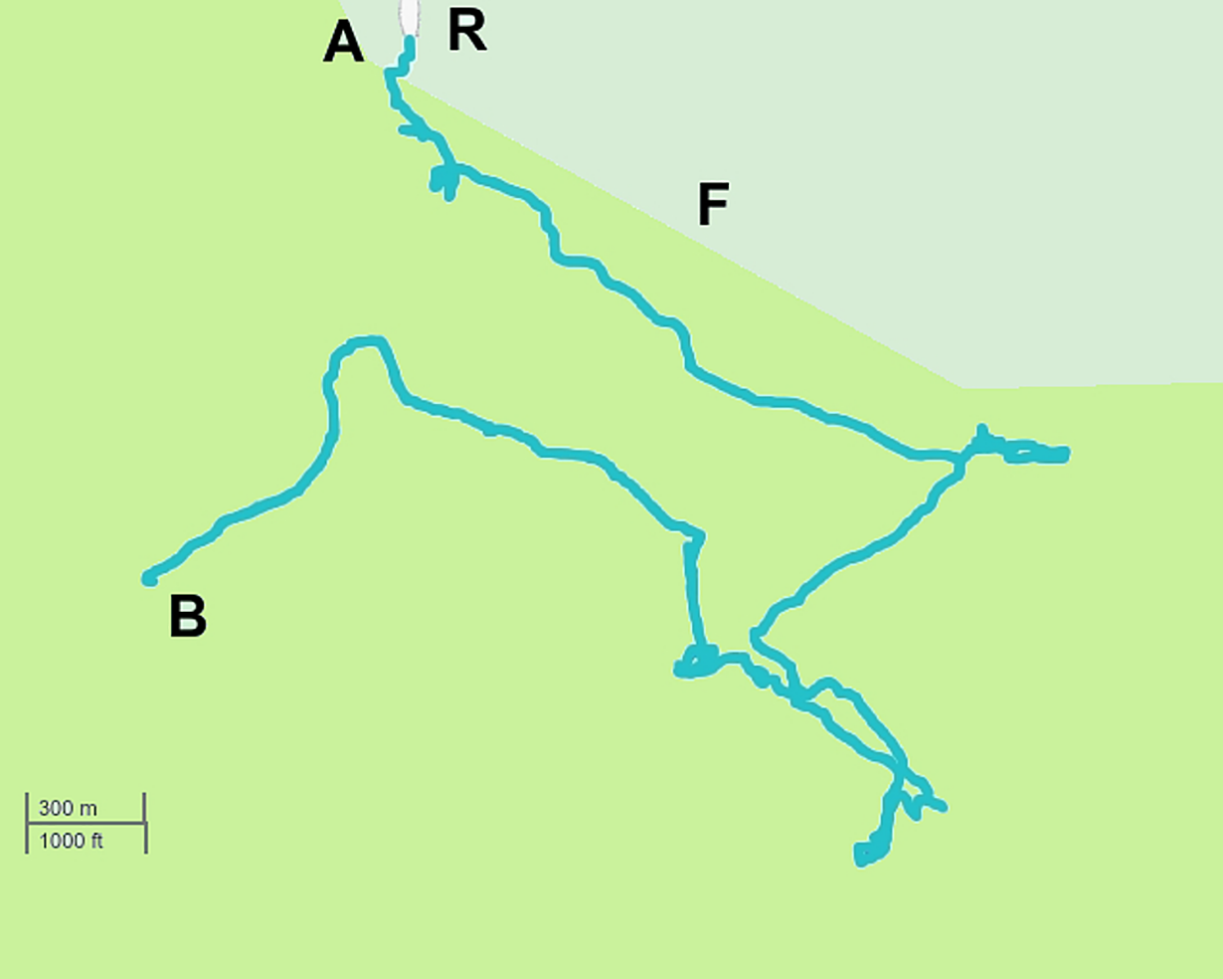
Trail following by the Brazilian Cariri gatherers. Typical example of a GPS recorded trajectory followed by an individual while executing a fruit gathering exploration in the Chapada do Araripe, Brazil. The trajectory starts at the end of a dirt road/trail and most of it follows the contours of a cliff edge (F), marked as the boundary of the two green colors. The foraging was made in the top of the mountain were the natural park is located. A is the start of the walking trajectory while B marks its end. R are dirt roads. There is also an extensive network of a footpaths. Location coordinates: 7°23'11.60"SS, 39°24'35.82"W. Map elaborated from our own GPS records. See S1 File for more details about this figure.

**Fig 6:**
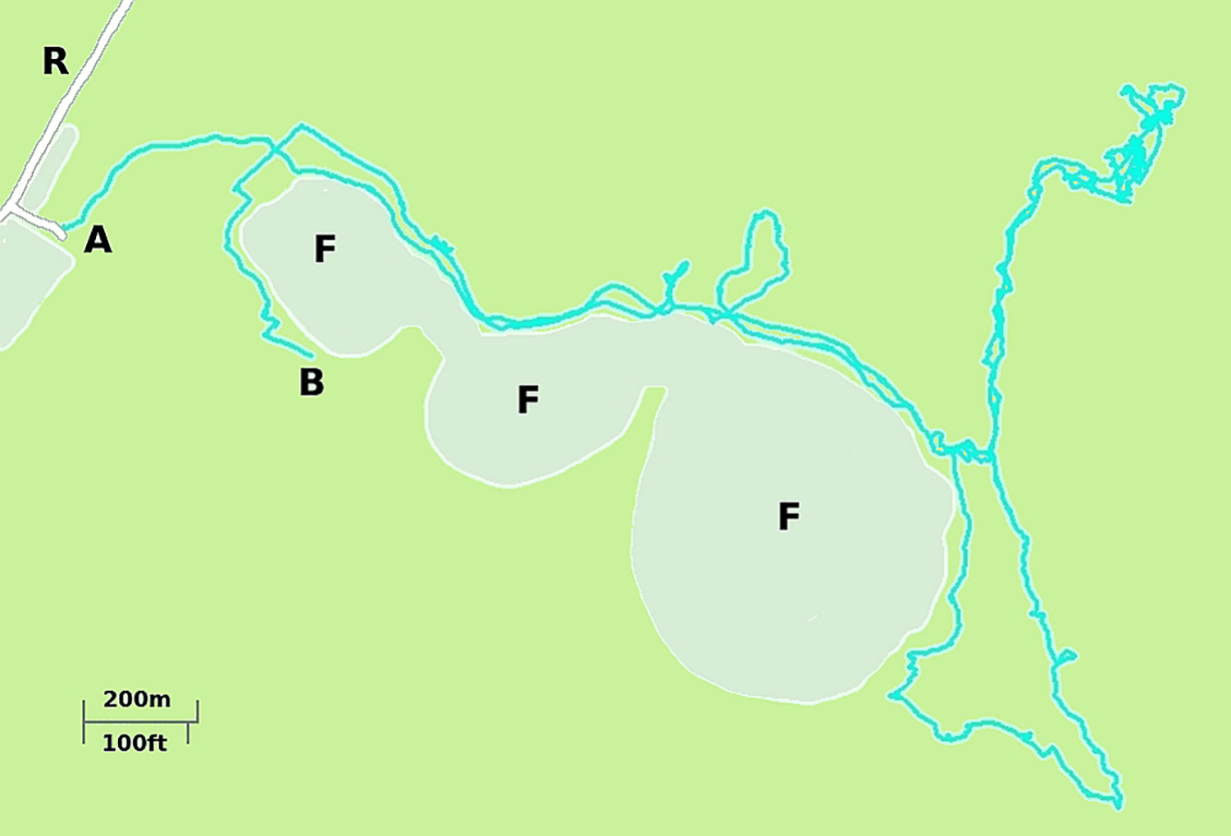
Typical example of a GPS recorded trajectory followed by an individual while executing a nut gathering excursion in the Amazonian rainforest in Rondônia, Brazil exhibiting the local terrain features as well. In some cases forest features (i.e. changes in canopy density) other than footpaths are used when walking, these may include the borders of forest gaps where vegetation is secondary or primary (F) and is somewhat more easy to walk. A is the start and B the end of the trajectory, R is a dirt road. Location coordinates: 10°53'40.68" S, 65°03'44.68"W. Map elaborated from our own GPS records. See S1 File for more details about this figure.

**Fig 7:**
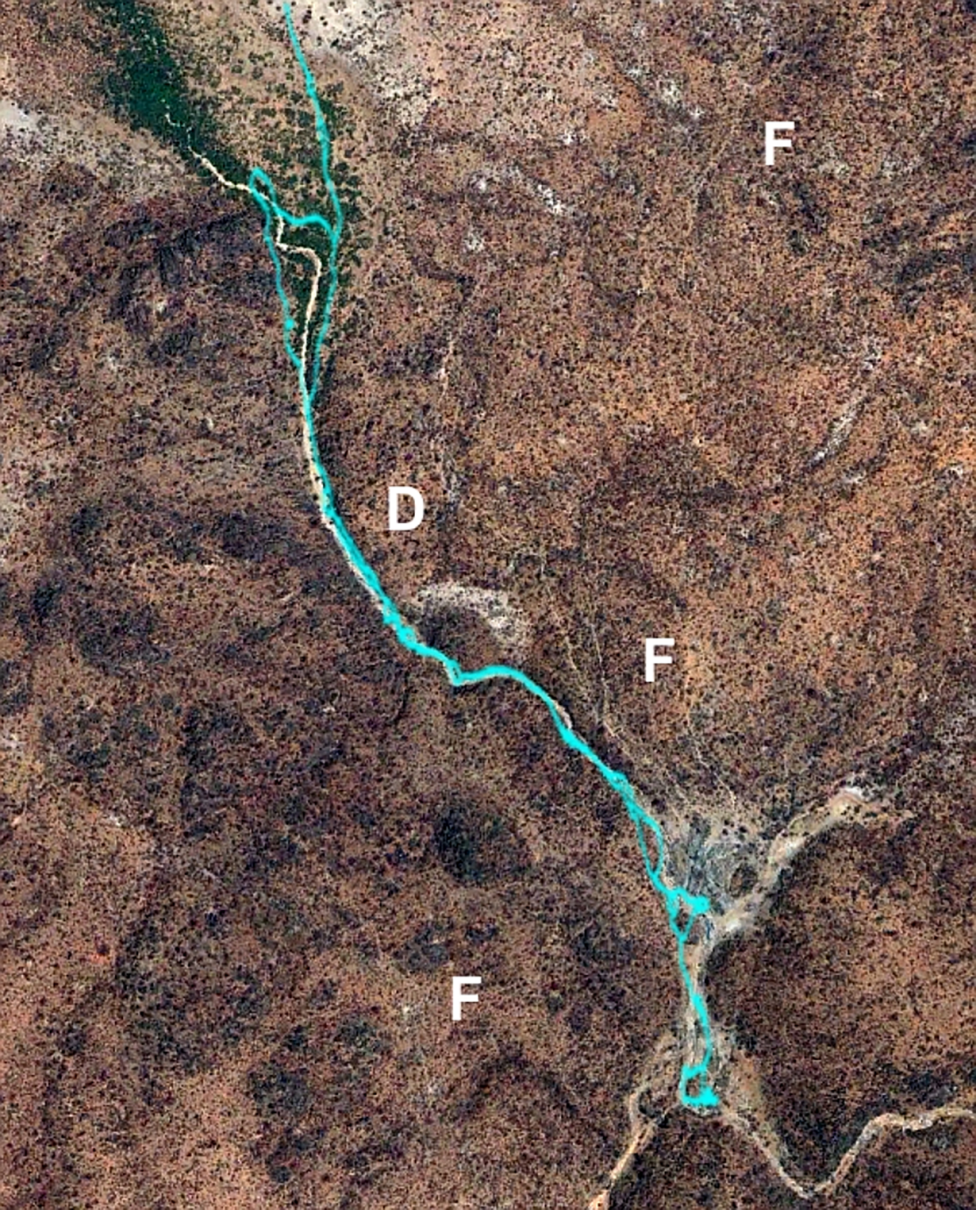
Feature following by the Hadza. A GPS recorded trajectory followed by an individual while executing a foraging trip, south of lake Eyasi in the Arusha region of central Tanzania, and overlaid on the local landscape (trajectory in blue from graph 2a in Raichlen et al, 2014). Location coordinates: 3°43'32.70"S, 35°11'7.00"E. In the image, the individual follows terrain features such a dry river basin (D). A network of footpaths is also evident (F). It remains to be seen whether or not feature and/or trailing following is evident in any other of 341 Hadza recorded trajectories. Image source is DigitalGlobe Inc. under a CC BY 4.0 license.

We found that around 50% of the search patterns resemble Lévy walks typically characterised by power-law (Lévy exponents) close to 1.7 and around 50% of the search patterns more closely resemble bi-phasic walks (Figs 1–3). The Lévy walks and bi-phasic walks can, however, provide very similar fits to the data and visually the fits are sometimes indistinguishable. There is no support for tri-phasic walks. Lévy walks typically characterised by power-law (Lévy) exponents, *μ*, around 1.7 were found to occur in similar abundance in the search patterns of the Hadza [9]. (But note that in [9] steps were determined by two kinds of events, turns and pauses rather than just by turns which break directional persistence). The remaining search patterns more closely resemble multi-phasic walks. We also find that the farmers are frequently walking on or close to pre-existing trails (Figs 4–6 and S1 File). This juxtaposition of Lévy walk characteristics and trail/feature following is evident, at least on one day (the exemplar in [9]), in the Hadza (Fig 7). However, it remains to be seen whether trail-following is common in the Hadza.

## Some speculative theories awaiting experimental verification

At first glance there might seem to be a clear contradiction between Lévy walk foraging patterns and trail/feature following as the trails/features are not likely to be shaped in ways that will result in Lévy walk patterns for trail/feature followers. These movements can, nonetheless, arise in a variety of ways. Gravity, for instance, influences water and it influences people, often leading to similarities in the courses of rivers and the paths that energetically optimal paths follow. Least cost paths often correlate with, or follow, natural features of the landscape, avoiding elevation gains. If the trails were to follow contour lines, which connect point of equal elevation (least effort); ridge tops (drainage divides); or streams in the valley then they may be fractal, and so result in Lévy-like movement patterns. This is because contour lines are analogous to coastlines which are known to have fractal properties. Indeed, the coastlines of Great Britain and Norway as well as a variety of contour lines across the United States are reported to be characterized by fractal dimensions ranging between about 1.2 and 1.5 [24–26]. But such fractal scaling seems not to account for our observations because Lévy walks have fractals dimension *μ-1* (i.e., fractal dimensions around 0.7) [27] and, more pertinently, because the Me’Phaa are foraging over (rather than around) hilly terrain [19] whilst the Cariri and Amazon people mostly forage over flat terrain.

Lévy-like movement patterns can also emerge from deterministic searching behaviours, as first suggested by Boyer et al. [28] who attributed Lévy-like movement patterns in the spider monkey, *Ateles geoffroyi*, to the use of cognition and mental maps.

**Fig 8:**
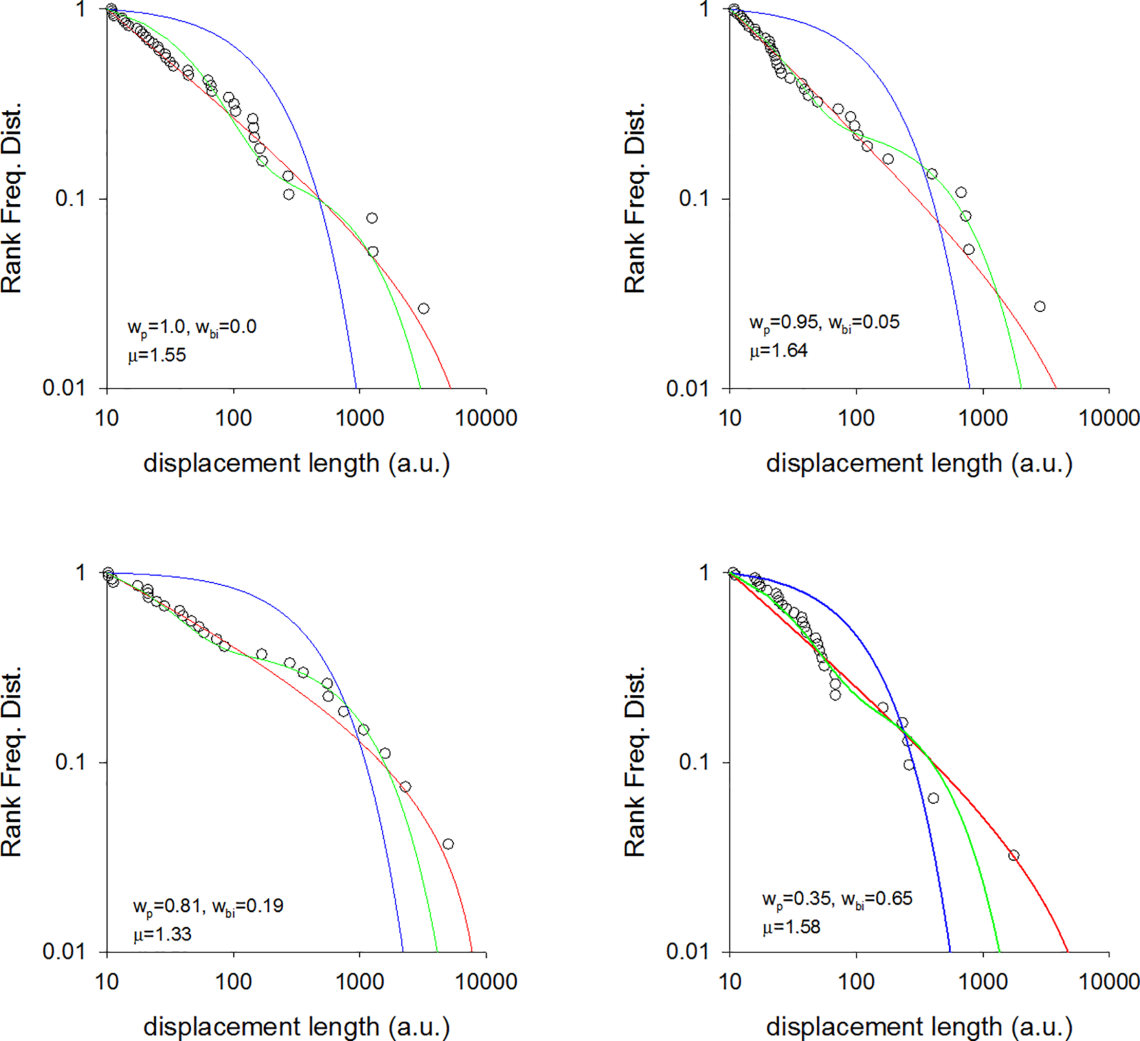
Fittings to the step length distribution for 4 simulated individuals. Rank frequency plots of the step lengths are shown (o) together with fits to power-law (red-lines), exponential (blue lines) and bi-exponentials (green lines). The Akaike weights, w_p_ and w_bi_, for the power-law and bi-exponential fits are shown together with the maximum likelihood estimates for the power-laws (Lévy) exponent, μ. Individuals perform random walks when making excursions. There is a single, straight trail. The walker moves with constant speed parallel to the trail and moves randomly in the orthogonal direction. Step-lengths in these random walks are exponentially distributed with mean length 1 a.u. An excursion ends when the walker first returns to the trail.

We conjecture that a potential parsimonious explanation for our findings is that the foragers are frequently making excursions from the trails and that these excursions take the form of random walks (coming back to the trail). We do not have direct evidence for such excursions but we suspect that they do occur being triggered by opportunistic foraging, curiosity about the surroundings, or because the farmers have temporally lost contact with the trail. If the farmers tend to drift in the direction of the trail when making such excursions then the distribution of net displacements made during long excursions will have a 3/2 power-law tail. This is a consequence of the Sparre Andersen theorem [29,30]. Net displacements made during short excursions will be distributed differently having tails that deviate from 3/2 power-laws. The projection method, used in our data analysis, would identify these net displacements made parallel to the trails if directional persistence were momentarily broken whenever farmers re-joined the trails. Random walking in both the parallel and orthonormal directions can, however, result in displacement distributions with inverse-square power-law tails [31]. Data from numerical simulations reveals that longer excursions comply with the dictates of the Sparre Andersen theorem [29,30], as do some shorter excursions. Estimates for the characteristic power law exponents (Fig 8) tend to fall between 3/2 and 2 in accordance with our observations (Figs 1–3) and our theoretical expectations. Other excursions more closely resemble bi-phasic walks (Fig 8) in accordance with our observations (Figs 1–3). Moreover, the prevalence of Lévy walks and adherence to the dictates of the Sparre Andersen theorem increases as the tortuosity of the excursion increases, leading to expectation that the emergence of Lévy walks may not be random but rather associated with certain tasks. There is, in fact, some indication that the median Lévy exponents reported for the Cariri fruit gatherers (Fig 2) are different from those of the nut gatherers (Fig 3) (Mann-Whitney U = 0, z = -2.087, p < 0.04).

An alternative but associated explanation for our observations is related to the fact that an individuals’ propensity, *p*, to move forward along a trail may change over time because of changes in an individuals’ internal state and because of changes in external environmental factors. In this case the distribution of steps taken along a trail between consecutive departures from it will be given by

$$P(n)=\int_{0}^{1}p^{n}(1-p)F(p)\mathrm{dp}$$ (1)

where *F*(*p*) is the distribution of propensities, *p*, whose functional form is unknown as the subject’s actual psychological propensities were not measured. People learn, have memory and use heuristics to judge probabilities and risks, and their motivations are often complicated, difficult to decipher, and obscure. In the absence of any knowledge about an individual’s motivations and behaviours the least biased choice for *F*(*p*) is the non-informative prior which is a uniform distribution when the propensities, *p*, are discrete and Jeffreys prior, $F(p)=\frac{1}{\pi \sqrt{p(1-p)}}$, when they form a continuum [32]. [In Bayesian statistical inference, a prior probability distribution, of an uncertain quantity is the probability distribution that would express one's beliefs about this quantity before some evidence is taken into account]. The use of any other priors would effectively amount to assuming more information about the motivational states and behaviours of the foragers than is known from the data. Notice that Jeffreys’ prior captures the tendency for individuals to either move along trails or search intensively (turn frequently) because it favours small and large propensities over middling propensities.

With this specification $P(n)=\frac{\sqrt{\pi }}{2}\frac{\Gamma (n+3/2)}{\Gamma (n+4)}\vec{n>>1}\frac{\sqrt{\pi }}{2}n^{-3/2}$ which is the hallmark Lévy-like characteristic seen in our data for Mexican and Brazilian farmers and in the movement patterns of the Hadza [9]. More generally only a few propensities, *p*, will be realized during a foraging trip so that the actual step-length distribution will be given by $P(n)=\sum_{i=1}^{N}p_{i}^{n}(1-p_{i})$ where the *N* realized propensities, *p*_*i*_, are drawn at random from *F*(*p*). The results of numerical simulations (not shown) reveal that some of these step-length distributions resemble bi-exponentials whilst others more closely resemble power-laws typically with power-law (Lévy) exponents, *μ*, close to 3/2. Furthermore, simulated movement patterns resembling Lévy walks with μ>2 are noticeably absent in accordance with our observations and with a previous analysis of the Hadza foraging patterns [9]. These predictions are the most conservative, maximally non-committal expectations for opportunistic and occasionally inquisitive trail/feature-following foragers. Models making other predictions would implicitly be making further assumptions about searching behaviours. Moreover, the theories based upon the Sparre Anderson theorem and upon Jeffrey’s Prior, although different from the mathematical perspective, have the same physical content and so are complementary rather than competing models. They thereby offer two complementary avenues for model development and refinement.

Aside from the aforementioned alternative explanation which invoked Jeffrey's prior, there could be other possible explanations for the occurrence of Lévy walking in rural farmers. They could, for example, be an emergent property of moving within a fractal landscape [33] or an emergent property of simple heuristics [34]. According to the analysis of Isliker and Vlahos [30] Lévy walks with *μ = 3/2* would emerge in foragers that randomly re-orientate after encountering objects (distractions or potential resources) whose spatial distribution is fractal with fractal dimension ½. To the best of our knowledge such objects have not been reported on. Wiens and Milne [35] did, however, report that the mosaic structure of vegetative cover in semiarid grasslands is fractal with fractal dimensions of 1.85 and 1.89. Heuristics are the psychological mechanisms by which people judge probabilities, frequencies, risks, and availabilities. Namboodiri et al. [34] reported that one such heuristic, temporal discounting, can provide selection pressures (but not a mechanism) for Lévy walking during exploration phases when cognitively sophisticated foragers are attempting to require information about the spatial distribution of resources. This framework, which was later elucidated by da Luz et al. [36], predicts *selection* for Lévy walks with *μ = 3/2* when the duration of the exploitation phase is capped [Reynolds, unpublished derivation]. But this, in itself, is not sufficient to explain our findings because the temporal discounting framework *presupposes* that individuals have the propensity to Lévy walk and therefore leaves open the key issue of identifying putative generative mechanisms for these Lévy walks. A prime candidate mechanism, consistent with trail following, would be meandering (i.e., the Sparre Andersen Theorem), making the difference between the predictions of our theory and that of Namboodiri et al. [34] difficult to discern, and making the two theories mechanistically equivalent to each other. Nonetheless, there is no problem with equifinality because Sparre-Anderson theorem is mechanistic whereas temporal discounting is not. More speculative is the possibility that the vegetative margins which the foragers sometimes follow are "percolation boundaries" marking the places where one vegetative species is invading another. Percolation boundaries have similitude with self-avoiding walks which in turn have similitude with Lévy walks [37,38]. Thus, following vegetation margins could, in principle, lead to movement patterns resembling Lévy walks. But this cannot explain our observations because such Lévy walks have μ~2.3 [38].

## Discussion

It has become clear that movement patterns resembling Lévy flight patterns occur widely across taxa [1–15] and frequently in scenarios seeming to fall outside of the “Lévy flight foraging hypothesis” [16,17] and the incumbent notion of optimal probabilistic searching. The identification of Lévy walk movement patterns in the Hadza is perhaps the most striking and important example of Lévy walking that cannot be attributed to optimized probabilistic searching, as humans have sophisticated cognitive abilities and so can return to locations where resources were found previously. Here we showed that the Hadza are not alone in this regard, as the Me’Phaa, the Brazilian Cariri and Amazonian farmers perform the same kind of Lévy walks. This apparent universality seems remarkable given that human behaviours are strongly shaped by an individual’s psyche and by complex social and environmental interactions, and given that “humans are the most cognitively complex foragers on Earth” [9]. These findings could have important implications for understanding human movement in both the present and the past [9]. It is also suggestive of an important link between the foraging patterns of humans and non-human animals [9].

These important issues can only be addressed once the underlying generative mechanism giving rise to the Lévy walks has been identified. Lévy-walk movement patterns could, in principle, result from non-random foraging behaviours when intelligent individuals make heavy use of cognitive properties such as mental maps, memory and complex decision making when dealing with previously-known environmental heterogeneities, which is especially true of primates and humans [28,39,40]. Here we showed that the movement patterns of the Me’Phaa, the Cariri and the Amazonian farmers are consistent with theoretical expectations for rural humans that tend to follow trails, often generated and reinforced to connect two sites following local optimality principles [41], and to occasionally stray from these trails either because they lose their way or because they engage in exploring their local surroundings; which is common in humans [42,43]. Our evidence comes from [1] the observed tendency of the rural humans to follow trails (Figs 4–7, and S1 File), [2] the juxtaposition of directed Lévy walks and bi-exponential walks that are consistent with [3] the Sparre Anderson theorem and with data from numerical simulations of meandering foragers (Fig 8). We identified and discounted some other candidate mechanisms. If our interpretation is correct then the occurrence of Lévy walk movement patterns (along with bi-modal movement patterns) in rural humans is no more mysterious than is their occurrence in the wandering albatross, which can be attributed to the birds following ephemeral odour trails [14]. Nonetheless, our interpretation is certainly not proven because we do not have detailed observations of meandering. It could therefore be confirmed or refuted by observations made at smaller scales.

## Permission to carry out fieldwork

All field activities were carried were approved by the relevant civil organizations acting on behalf of the volunteers. In Mexico this was the Xuajin Me'Phaa A.C. In Brazil it was: Associação de Moradores de Cacimbas (município de Jardim–CE); Associação dos Pequenos Produtores do Sitio Macaúba (município de Barbalha–CE); Associação dos Seringueiros da Reserva Extrativista do Rio Ouro Preto (município de Guajará-Mirim- RO); Associação dos Seringueiros Agroextrativistas do Baixo Rio Ouro Preto (município de Guajará-Mirim- RO); and the Comitê de Ética de Pesquisa em Humanos da UERN (Universidade Estadual do Rio Grande do Norte). In the case of research carried on public lands in Brazil, the project was reviewed and approved by ICMBio, the Chico Méndez Institute for Biological Diversity.

## Supporting information

S1 File Link to satellite images corresponding to Figs 4, 5 and 6 together with further examples of recorded trajectories in Mexico and Brazil. An example of a simulated trajectory (Figure A). Example of a simulated trajectory. At each time-step incremental displacements in the direction of the trail (running east to west) were drawn at random from an exponential distribution with mean 1 (arbitrary units a.u.). Incremental displacements in directions orthogonal to the trail made during excursions were also exponentially distributed with mean 1 (a.u.). The distribution of the lengths of the excursions has a 3/2 power-law tail.
(DOCX)

S2 File Fortran code used to fit model distributions to GPS data. (FOR)

S3 File Fortran code to simulate meandering and showing emergence of Lévy walks. (FOR)

S4 File GPS data for the Amazonian farmers. (ZIP)

S5 File GPS data for the Brazilian Cariri farmers. (ZIP)

S6 File GPS data for the MePhaa. (ZIP)
