# Supplementary material for: Lévy foraging patterns of rural humans
Source: PLoS One. 2018 Jun 18;13(6):e0199099. doi: 10.1371/journal.pone.0199099 (PMC6005560; doi:10.1371/journal.pone.0199099)
Supplement: S1 File — An example of a simulated trajectory (Figure A). Example of a simulated trajectory. At each time-step incremental displacements in the direction of the trail (running east to west) were drawn at random from an exponential distribution with mean 1 (arbitrary units a.u.). Incremental displacements in directions orthogonal to the trail made during excursions were also exponentially distributed with mean 1 (a.u.). The distribution of the lengths of the excursions has a 3/2 power-law tail. (DOCX) [file pone.0199099.s001.docx]

**Supplementary Information**

Satellite images corresponding to Figures 4,5 and 6 together with further examples of recorded trajectories in Mexico and Brazil are available at the Harvard Dataverse at the following link:

https://doi.org/10.7910/DVN/GR121L





**Figure A**
